# Association between continuous device-based physical activity monitoring over six months and cardiorespiratory, metabolic and body composition outcomes in post-surgery cancer survivors - An observational cohort analysis within a randomized controlled trial (CRBP-TS Study)

**DOI:** 10.1186/s13102-025-01275-3

**Published:** 2025-08-09

**Authors:** Christian Leps, Christian Bischoff, Ines Gockel, Uwe Tegtbur, Stefan Kwast, Christoph Pökel, Johannes Voß, Hans-Jürgen Rinser, Roberto Falz, Martin Busse

**Affiliations:** 1 Institute of Sports Medicine and Prevention, University Leipzig, Leipzig, Germany; 2 Department of Visceral, Transplant, Thoracic and Vascular Surgery, University Hospital Leipzig, Leipzig, Germany; 3 Institute of Sports Medicine, Hannover Medical School, Hannover, Germany; 4 Helios Health Institute, Berlin, Germany; 5 Quadecco GmbH, München, Germany; 6 Human-Machine Interaction, Magdeburg-Stendal University of Applied Sciences, Magdeburg, Germany

**Keywords:** Consumer wearable device, Physical activity, Cardiorespiratory fitness, Biomarkers, Cancer survivor

## Abstract

**Background:**

The current guidelines for physical activity often rely on self-reported data or short-term activity tracking. We aimed to explore device-based long-term physical activity tracking and its possible association with cancer survivors’ cardiorespiratory fitness (CRF), metabolic health, and body composition.

**Methods:**

In this observational analysis of a randomized controlled trial (CRBP-TS study), we reanalyzed data from 111 patients with breast, prostate, and colorectal cancer. Dependent variables included cardiorespiratory outcomes, body composition, metabolic biomarkers, and fatigue. A multiple linear regression model was used to analyze the data, considering age, gender, BMI, and baseline values. A consumer wearable device measured moderate-to-vigorous physical activity (MVPA) and steps continuously over six months, ensuring a detailed and accurate record of the participants' daily physical activity.

**Results:**

Physical activity data from all participants indicated a mean of moderate physical activity 108 min/wk (SD ± 88), vigorous physical activity 41 min/wk (SD ± 36), and 8498 steps/day (SD ± 2490). We observed that higher levels of MVPA were significantly associated with higher maximum oxygen uptake (VO_2max_; β = 0.5, 95% CI [0.02 to 1.0], *p* = 0.042), higher peak power output (PPO; β = 0.04, 95% CI [0.003 to 0.08], *p* = 0.037), and higher cardiac output (β = 0.6, 95% CI [0.2 to 1.1], *p* = 0.009). Additionally, more steps correlated significantly with higher VO_2max_ (β = 0.27, 95% CI [0.04 to 0.51], *p* = 0.023), higher cardiac output (β = 0.2, 95% CI [0.013 to 0.47], *p* = 0.039), lower fat mass (β= -0.24, 95% CI [-0.44 to 0.03], *p* = 0.028), lower insulin (β=-4.2, 95% CI [-6.4 to -2.0], *p* < 0.000), and lower leptin (β=-0.56, 95% CI [-0.97 to -0.15], *p* = 0.008).

**Conclusions:**

Continuous activity tracking with wearable devices provides an objective and standardized opportunity to investigate the amount of aerobic physical activity and its association with systemic health outcomes in cancer survivors. Our long-term activity data support a positive relationship between aerobic physical activity and cardiorespiratory fitness as well as metabolic health.

**Trial registration:**

DRKS-ID: DRKS00020499; *Registered 17 March 2020*, https://drks.de/search/en/trial/DRKS00020499.

## Key points

• A positive association exists between MVPA or steps and higher VO_2max_ and cardiac output in cancer survivors.

• An inverse association exists for additional steps and lower insulin and leptin metabolic biomarkers in cancer survivors.

• Consumer wearable devices are a suitable measurement method for objectively quantifying the effects of long-term aerobic physical activity on health outcomes.

## Background

In 2022, there were close to 20 million new cancer cases and 9,7 million cancer deaths [1]. Substantial evidence supports that there is an inverse relationship between cardiorespiratory fitness (CRF) and cancer-related mortality [2, 3]. Physical activity improves cancer prognosis and survival, especially in breast cancer, prostate cancer, and colorectal cancer [4]. The current WHO physical activity guidelines recommend a target range of 150–300 min of moderate-intensity or 75–150 min of vigorous-intensity physical activity per week or an equivalent combination of moderate-intensity and vigorous-intensity aerobic physical activity for people with chronic diseases or disabilities, whereby each minute has a health effect [5]. The impact of physical activity on cancer metabolism involves suspected molecular mechanisms that affect cellular nutrient availability and systemic outcomes such as body weight, energy balance, endocrinology, microbiome, inflammation, and immunity [6, 7]. Despite general physical activity recommendations, there is a lack of the use of wearable devices throughout the entire study period to establish a temporal relationship between physical activity and metabolic or inflammatory markers, body composition, and cardiorespiratory capacity in cancer patients.

A significant limitation arises from the differences in the generation of physical activity data. Researchers usually have to rely on subjective self-reported physical activity data [8]. Relying on self-reported physical activity data can result in inconsistencies and lead to flawed conclusions when making recommendations [9]. Therefore, the measurement method (subjective vs. objective) significantly impacts the observed magnitude of physical activity. In addition, the differences between self-reports and device-based measures appear to increase with vigorous physical activity (VPA), distorting the interpretation of VPA [10]. In conclusion, the variation in data collection methods is a significant challenge to the recommendations for aerobic physical activity. Wearable devices represent a promising approach to measuring physical activity in an objective and standardized way [11].

Medical research studies have used consumer-based wearable devices to record aerobic physical activity [12]. Wearing consumer-based wearable devices already increases physical activity in healthy individuals and those with chronic diseases [13, 14]. Breast cancer survivors have found activity trackers helpful for following physical activity recommendations, and the device’s design can influence their motivation to wear them [15]. In general, wearable technology presents an inexpensive and scalable opportunity to facilitate more active lifestyles for cancer survivors [16]. In addition, the association between physical activity and cardiometabolic and endocrine improvements was more significant in accelerometer-measured physical activity compared to questionnaire/self-report [17].

Consumer wrist-worn devices with acceleration and heart rate sensors can measure steps and MVPA via different sensors. These wearable devices frequently use acceleration sensors for step data generation and have acceptable accuracy [18, 19]. Non-step-based parameters, especially energy consumption and heart rate, have low to moderate validity for consumer-based wearable devices [18]. However, despite the different validation methods and a lack of standardization, a meta-analysis concluded that consumer wearable devices are valid for measuring MVPA [20].

This paper is based on data from the randomized controlled CRBP-TS trial that investigated the effectiveness of home-based strength-endurance training for cancer patients after surgery. We analyzed six months of continuously tracked aerobic physical activity (including leisure and occupational exercise) with consumer wearable devices. This allows us to reflect an accurate estimation of physical activity behavior over a significantly more extended period than previously considered. It re-evaluates the relationship between daily aerobic physical activity and systemic health outcomes, including cardiorespiratory fitness, hemodynamics, body composition, biomarkers, fatigue, and cancer-specific quality of life (QoL), in cancer survivors. We hypothesized that a higher level of MVPA and steps would be associated with significantly improved systemic health outcomes.

## Methods

### Study design

This manuscript reports a secondary cohort analysis from a prospective randomized controlled trial (CRBP-TS Trial). The study protocol for CRBP-TS was described in detail in 2021 [21] and the main results were reported in 2023 [22, 23]. CRBP-TS was a randomized, controlled, multicenter (Dresden, Hannover, Leipzig, Germany) trial to implement a multimodal, home-based, and individually adapted online training program to improve physical activity and performance. A consumer wrist-worn device tracked aerobic physical activity (MVPA and steps) continuously throughout the intervention period (six months) in the intervention group (IG) and control group (CG). The intervention group additionally engaged in home-based exercise bodyweight training (30 min per session) with help from videos via an App with alternating strength-endurance parts, information on general health improvement, disease prevention, and lifestyle changes (diet, exercise, and self-awareness) from the study team (physician, sports scientist, and a study nurse) via the app. Subjects in the original intervention group were advised to increase their overall physical activity in addition to their home training intervention.

The present manuscript’s observational analyses were not part of the original CRBP-TS study design. The CRBP-TS study was approved by the Ethics Committee of the Medical Faculty, University of Leipzig (reference number 056/20-ek), as well as at all participating sites. The study was registered at DRKS with trial ID DRKS00020499, and the first registry was on March 17, 2020.

### Participants

The CRBP-TS study enrolled participants who were cancer patients applying the international classification of diseases (ICD) Codes C18/19/20 (colorectal cancer), C50 (breast cancer), and C61 (prostate cancer) in those who had undergone curative (R0) surgery at stages T1N0M0 to T3N3M0 (tumor, node, metastases); ECOG (Eastern Cooperative Oncology Group) ≤ 1; and were between 18 and 75 years of age.

Both groups, the intervention and control group, got structured information on general health improvement, disease prevention, and lifestyle changes (diet, training, and self-perception) from the study team (physician, sports scientist, and a study nurse). Additionally, all patients in both the intervention and control group received activity feedback via wearable devices. The intervention group performed the home training and the recommendations for two (at least) or preferably three times or more per week for 30 min of home-based and bodyweight online endurance-resistance training. Additionally, various measures were implemented to increase physical activity, including activity biofeedback through an app and tablet, structured information, training reminders, and assessments to raise awareness of individual fitness levels. Due to the distance-based training setting, there was no immediate control over whether the weekly training recommendations were implemented. However, this was a realistic setting that reflects the life situation of most people.

**Fig. 1:**
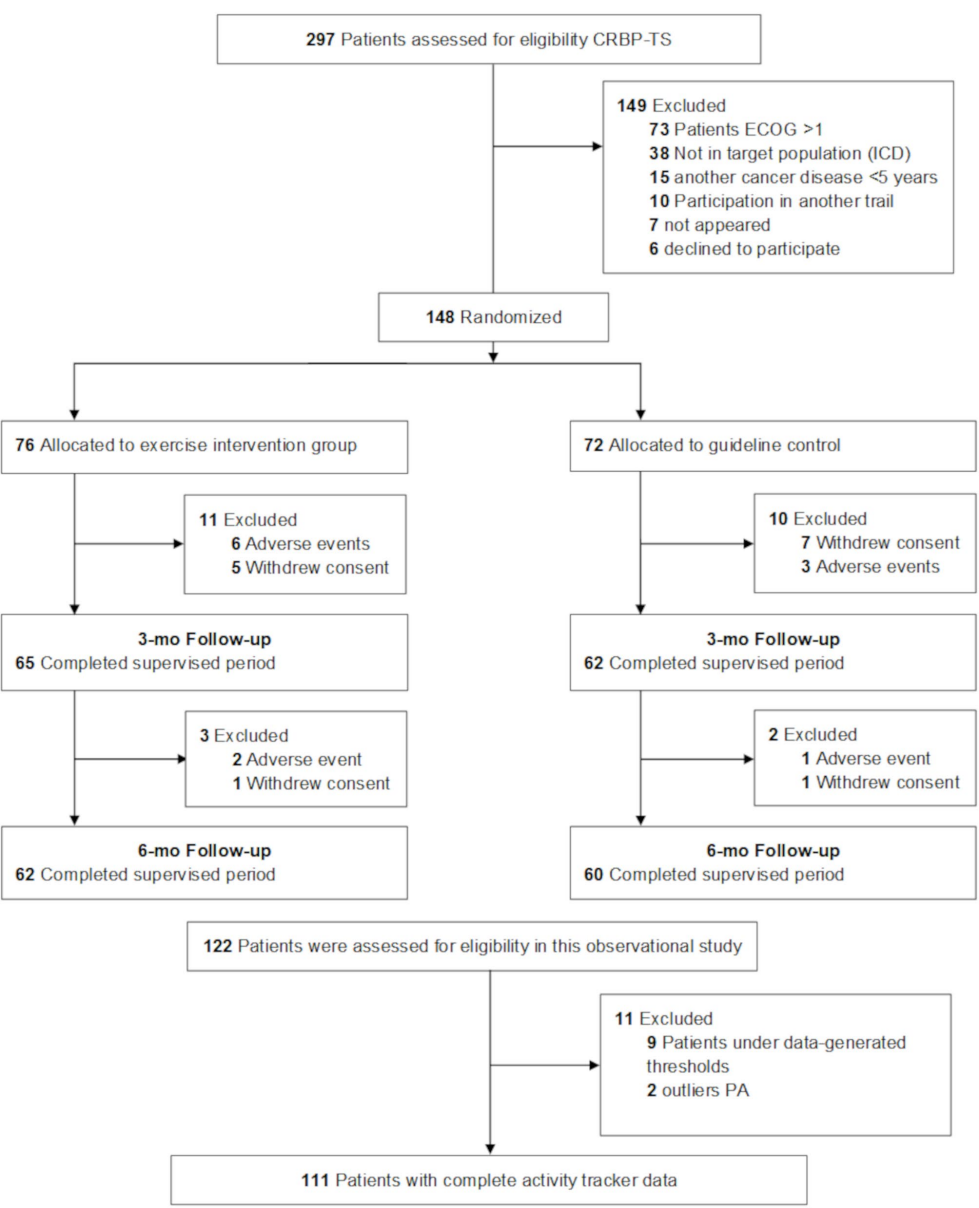
CONSORT Flow diagram

Researchers have contended that a cancer diagnosis may function as a ‘teachable moment’ in which a cancer survivor’s motivation for lifestyle change may be incredibly high [24]. We assume, and the data from the wearable device support this, that the control group was also physically active (rehabilitation sports groups, gym, etc.). The two groups showed no significant differences in physical activity. The intervention group indicated a mean of steps 8430 per day (SD ± 2560), MPA 113 min/wk ((SD ± 101), and VPA 40 min/wk (SD ± 31), and the control group a mean of steps 8570 per day (SD ± 2437), MPA 102 (SD ± 73) and VPA 42 (SD ± 40). The wearable devices enabled us to record each participant’s complete aerobic activity profile, regardless of their group affiliation. Despite the interventional patients in the original CRBP-TS trial participating in an intervention home training program that affected physical capacity (VO_2max_), the control group patients revealed steps and MVPA closely resembling those of the patients in the IG, which is why we analyzed the activity data from both study groups with completely 111 participants (Fig. 1). In this observational study, we included only those participants from the intervention group and control group, who met the recommended minimum for thresholds for data generation of device based physical activity (see next section Activity tracking/Exposure).

In the original CRBP-TS study, 18 adverse events in 16 patients (11%) were documented and classified as serious adverse events (SAE) that were unrelated to the exercise intervention (no temporal relation to training, other cause such as accident, new disease diagnosis, or scheduled surgery). 12 of the 16 patients were excluded due to incomplete activity data and were not included in this observational analysis.

### Activity tracking/Exposure

All participants were given a consumer wrist-worn wearable device at baseline (vívoactive 4 and 4s; Garmin, Olathe, Kansas, USA) to determine daily steps with an acceleration sensor and MVPA with a heart rate sensor. The Garmin wearable devices calculate MVPA by comparing the current heart rate to the average resting heart rate, whereby age, weight, and height are also taken into account in the calculation [25]. In conclusion, there was no interrelationship between steps and MVPA calculation.

The physical activity was measured continuously between the pre- and post-tests and was averaged over the entire study period; thereby, we could establish a temporal relationship between physical activity and outcomes. The minimum reporting thresholds for data generated by wearable activity trackers, which most studies require, were considered. A valid day is defined as having at least 10 h of wear time within a single day, and a valid interval is defined as a week with at least three valid days [12]. In our study, the wearable device was worn continuously for 24 h throughout the study period. A day was considered valid if data from more than 10 h of device-based activity was recorded during the awake period. A week was considered valid if it contained at least four valid days per week. The data were then normalized to a seven-day period, and weekly averages were calculated accordingly. We determined that each participant’s wearable device data was representative if it included at least 13 weeks of activity data. On average, participants had 22 valid weeks of data (SD: 3.6; min: 13; max: 26). Since the participants wore the wearable devices throughout the entire study period and were unaware of any data loss, we do not expect any changes in physical activity behavior during the weeks for which data was lost.

The number of minutes spent on aerobic physical activity per week in the moderate (3-5.9 METs) and vigorous (≥ 6 METs) intensity ranges was measured. In general, physical activity covers any activity enabled by increased energy expenditure, i.e., leisure-related and work-related activities [26]. Exercise and training are also muscular activities, but they are planned, structured, and performed repetitively to improve physical performance. In this respect, physical activity encompasses exercise and training as a subcategory, including all muscular activities in daily life. In the present analysis, daily MVPA or steps, rather than a specific exercise or training, served as exposure.

Physical activity recommendations in scientific studies are expressed in steps, active calories, MET minutes, MET hours, or MVPA. MVPA was created because MET minutes are too complicated for the non-scientific public [27]. However, all recommendations rely on an indication of 500–1000 active calories/MET min/wk to achieve a health benefit. 150 moderate activity minutes (3.3 METs x 150 min) or 75 vigorous activity minutes (6.7 METs x 75 min) are equivalent to the lower limit of 500 MET min/wk in EE. 300 moderate activity minutes (3.3 METs x 300 min) or 150 vigorous activity minutes (6.7 MET x 150 min) are equivalent to the upper limit of 1000 MET min/wk in EE. Vigorous activities thus count for twice as much as moderate ones [28]. In this study, we measured moderate and vigorous activity minutes, and the vigorous activity minutes were counted only twice to ensure comparability in calculating multiple linear regression models and active energy turnover.

Wearable device data were imported into the CRBP-TS application (App-Provider: DiaVention GmbH, Hannover, Germany) via a Bluetooth interface between the wearable and tablet (Lenovo Tab M10 TB-X606X; Lenovo, Hong Kong, China). The Garmin Health SDK (software development kit) transmitted daily data. Therefore, we got the activity data directly from the wearable devices via Bluetooth in our CRBP-TS application.

### Outcomes and clinical assessments

This observational analysis investigated the association between the dose of aerobic physical activity and primary and secondary outcomes of the CRBP-TS trial. The following parameters were included in our study:

- Cardiorespiratory fitness: We assessed the VO_2max_ (ml/kg/min), the change in VO_2max_ after six months was the primary endpoint in CRBP-TS, and the peak power output (Watts and Watts/kg). We used Dynostics Ergo-Spirometry, Sicada GmbH, Germany, to assess these parameters. The cardiorespiratory exercise was tested on an electronically braked semi-reclining ergometer. The starting load was 30 watts with a 10 watts/min increase until subjective or objective exhaustion or the occurrence of termination criteria [29]. Staff conducting the studies were not blinded to the treatment groups. Cardiorespiratory exercise capacity was assessed according to current recommendations and evaluated in a blinded manner at the study’s core laboratory in Leipzig, Germany. Maximum oxygen uptake (VO_2max_) was defined as the highest 30-second average within the last minute of exercise.
- Hemodynamic parameters: peak cardiac output (l/min) [CO] and adjusted rate-pressure product (HRxSBP) [RPP] were also measured during the cardiorespiratory exercise test. We used custo BT300 electrocardiogram, custo GmbH, Germany, and PhysioFlow impedance cardiography, Manatec Biomedical, France. Impedance cardiography is a reliable method for assessing hemodynamics during exercise and is sensitive to changes in cardiac function in patients [30, 31].
- Body composition: body weight, fat mass, and lean body mass (LBM). We used the BIACORPUS RX 4004 M, manufactured by MEDI CAL HealthCare GmbH, Germany, for phase-sensitive impedance analysis to measure body composition. BIA is a suitable and valid method for assessing body composition in oncology [32].
- Biomarkers: we analyzed the metabolic markers hemoglobin A1c (HbA_1c_), leptin and insulin. Blood parameters were analyzed in the central core laboratory (Institute of Laboratory Medicine, Clinical Chemistry and Molecular Diagnostics, University Hospital Leipzig, Leipzig, Germany).
- Psychological status: The European Organisation for Research and Treatment of Cancer core questionnaire with the module C-30 (EORTC QoL-C30) [33] and the Fatigue Severity Scale (FSS) were used to investigate cancer-specific QoL and fatigue.

### Confounder

For this observational study, we adjusted all analyses for variables deemed possible confounders for the six-month observational period. In the statistical multiple linear regression model, we included the following confounders:

- Age: metric variables calculated from the birth date until the date of study enrollment.
- Gender: dichotomy variable, male or female.
- Body-mass-index (BMI): metric variable.
- baseline: baseline values of the dependent variable, metric variable.

Other potential confounders were excluded from our analyses: The type of cancer (colorectal cancer, breast cancer, prostate cancer) would be a confounder, but is already taken into account by gender as a confounder, since only two females had colorectal cancer. As relevant *comorbidity*, only four participants had diabetes or cardiovascular disease and were excluded as covariates. No other comorbidities, such as orthopedic, rheumatological, or neurological disorders, were contraindicated for physical activity and training. Aerobic physical activity as exposure includes all daily activities, i.e., leisure, work, and exercise. We have previously reported no significant difference in the exposure to physical activity (steps and MVPA) between the intervention and control group over the intervention period. Since home training is only one component of the intervention, we believe it should not introduce significant bias beyond what is typically found in an observational study with comparable exposure and outcomes. As individual components of physical activity, particularly the type and amount of training, could not be fully known, we excluded the study group (intervention group vs. control group) as a confounder.

### Statistics

All participants providing sufficient aerobic physical activity data were included in our statistical evaluation. All analyses were conducted via linear models. Physical activity (MVPA or steps) was the exposure, and age, gender, BMI (excluded for body composition outcomes), and baseline values of dependent variables were adjusting covariates. We checked the following conditions to apply multiple linear regression: excluding extreme outliers (3 times the interquartile range) before the model calculation, examining the residuals for outliers after the analysis, normal distribution and independence of the residuals, linearity and homoscedasticity, and no multicollinearity between the variables. Our results are reported descriptively as the mean and standard deviation for baseline and after six months, as mean and 95% confidence intervals for moderate physical activity (MPA), vigorous physical activity (VPA), and steps, and as multiple regression analyses with adjusted R square, non-standardized Beta-Coefficient, 95% confidence intervals, and p-values. Data were analyzed using IBM SPSS Statistics (Version 29; IBM, Armonk, New York, USA) and displayed using GraphPad Prism (Version 9; GraphPad Software Inc., California, USA).

## Results

**Fig. 2:**
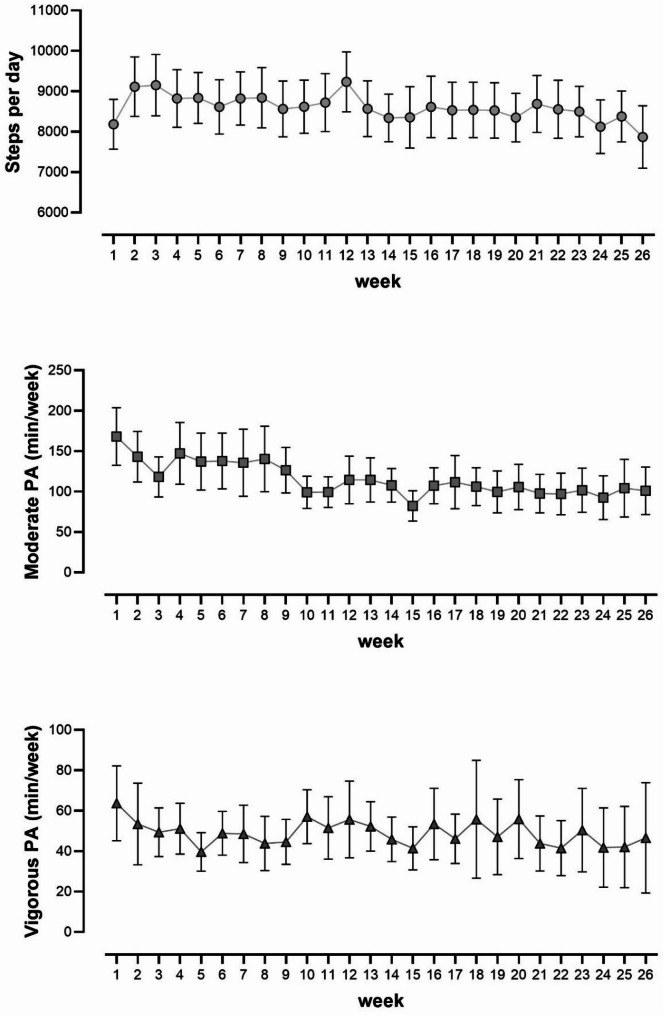
Steps per day, moderate physical activity, and vigorous physical activity in min per week during the observational period (*n* = 111; mean data recording 22 ± 3.6 weeks; presented as mean and 95% CI)

122 participants completed the CRBP-TS trial. The device-based physical activity data from 111 participants could be used for the present observational analysis (Fig. 1; Table 1). Sixty-seven participants in that population were females, of whom 65 were breast cancer survivors; only two females had colorectal cancer. The cancer entities were already accounted for by gender as a factor in the linear model. Table 2 presents the outcome variables (dependent variables) descriptively as a baseline and after six months with mean and standard deviations. The CRBP-TS study revealed no differences in MVPA or steps between the intervention and control group in the original research. The independent variable (exposure), physical activity for the total group (intervention group and control group), is presented as MPA (108 min/wk), VPA (41 min/wk), and steps (8498 per day) on average over six months. MPA, VPA, and steps are also presented as means and 95% confidence intervals for every week of the 26-week study period (Fig. 2).

The associations between MVPA or steps and cardiorespiratory fitness, body composition, biomarkers, fatigue, and cancer-specific QoL in cancer survivors after six months are illustrated in Table 3. A weekly increase of 100 min MVPA was significantly associated with higher cardiorespiratory fitness: VO_2max_ (ml/kg/min) (β = 0.5, 95% CI [0.02 to 1.0], *p* = 0.042) and peak power output (Watt/kg) (β = 0.04, 95% CI [0.003 to 0.08], *p* = 0.037), and higher cardiac output (l/min) (β = 0.6, 95% CI [0.2 to 1.1], *p* = 0.009).

For steps, a daily increase of 1000 was significantly associated with greater VO2max (ml/kg/min) (β = 0.27, 95% CI [0.04 to 0.51], *p* = 0.023) and higher cardiac output (l/min) (β = 0.2, 95% CI [0.013 to 0.47], *p* = 0.039). In terms of body composition, we observed negative relationship between fat mass (kg) (β= -0.24, 95% CI [-0.44 to 0.03], *p* = 0.028) and steps. Regarding metabolic biomarkers, highly significant lower insulin (pmol/l) (β=-4.2, 95% CI [-6.4 to -2.0], *p* < 0.000) and leptin (ng/ml) (β=-0.56, 95% CI [-0.97 to -0.15], *p* = 0.008) levels were associated with more steps after six months. Due to the low adjusted R-squared value for EORTC (Table 3), we were unable to assess cancer-specific QoL data in our linear model.

**Table 1:** Demographic and clinical characteristics

	Baseline, *n* = 111
Age, mean (SD)	54 (11)
BMI, mean (SD)	26.2 (4.5)
Sex, n (%)	
Female	67 (60.4)
Male	44 (39.6)
Cancer entity No., n (%)	
Colorectal cancer	12 (10.8)
Breast cancer	65 (58.6)
Prostate cancer	33 (29.7)
Comorbidities, No., n (%)	
Diabetes type 2	4 (3.6)
Hypertension	31 (27.9)
Obesity (BMI > 30 kg/m²)	23 (20.7)
Cardiovascular diseases	4 (3.6)
Hypothyroidism	23 (20.7)
Asthma	2 (1.8)
Arthritis	8 (7.2)
Depression	6 (5.4)
Cancer medication, n (%)	
Estrogen receptor modulator	19 (17.1)
Monoclonal antibody	2 (1.8)
Aromatase inhibitors	12 (10.8)
Chemotherapy medication	4 (3.6)

Values presented at the means, standard deviation, number and percentage. Abbreviations: BMI = body-mass-index

**Table 2:** Systemic health outcomes at baseline and after six months in cancer survivors, as well as mean physical activity over six months

	Baseline Mean (SD)	After 6 month Mean (SD)
Cardiorespiratory fitness		
VO_2max_, ml/kg/min	27.5 (6.0)	28.6 (6.6)
Peak power output, Watt	136 (34)	144 (39)
Peak power output, Watt/kg	1.80 (0.47)	1.90 (0.53)
Rate-pressure product	26,417 (5516)	25,877 (5320)
Cardiac output max, l/min	17.3 (3.25)	17.8 (3.56)
Body composition		
Body mass index (kg/m^2^)	26.1 (4.5)	26.2 (4.4)
Weight (kg)	77.4 (15.7)	77.6 (15.8)
Fat mass (kg)	23.0 (9.6)	22.4 (8.9)
Lean body mass (kg)	54.3 (11.0)	55.2 (12.1)
Biomarker		
CRP level (mg/l)	3.2 (12.0)	1.7 (1.8)
HbA_1c_ (%)	5.4 (0.5)	5.4 (0.4)
Insulin (pmol/l)	65 (49)	70 (80)
Leptin (ng/ml)	14.1 (17.4)	12.4 (13.3)
Quality of Life & Fatigue		
FSS	2.89 (1.60)	2.88 (1.59)
EORTC	62 (23)	70 (22)
Physical activity		
MPA (3–5,9 METs)	108 (88)	
VPA (≥ 6 METs)	41 (36)	
Steps (per day)	8498 (2490)	

Values presented at the means and standard deviation, Abbreviations: VO_2max_: maximum oxygen uptake; CRP = c-reactive protein; HbA1C = glycosylated hemoglobin; EORTC QLQ = European Organization for Research and Treatment of Cancer Quality of Life questionnaire; FSS = Fatigue severity scale; MVPA: moderate-to-vigorous physical activity; MPA = moderate physical activity; VPA = vigorous physical activity; MET = metabolic equivalent of task;

**Table 3:** Associations of MVPA and steps with health outcomes for cancer survivors. Presented as adjusted R-squared, unstandardized regression coefficient (Beta) with 95% confidence intervals and p-Value. The changes in outcomes relate to an increase in MVPA of 100 min per week or 1,000 steps per day

		MVPA			Steps		
	n	Adjusted R-squared	Beta (95% CI)	p-Value	Adjusted R-squared	Beta (95% CI)	p-Value
Cardiorespiratory fitness							
VO_2max_ (ml/kg/min)	108	0.81	0.5 (0.02 to 1.0)	0.042*	0.81	0.27 (0.04 to 0.51)	0.023*
Peak power output (Watt)	108	0.82	2.7 (0.1 to 5.3)	0.044*	0.82	1 (-0.2 to 2.4)	0.098
Peak power output (Watt/kg)	108	0.81	0.04 (0.003 to 0.08)	0.037*	0.81	0.02 (-0.002 to 0.04)	0.072
Hemodynamic							
Rate-pressure product	109	0.70	212 (-263 to 686)	0.378	0.70	-73 (-300 to 154)	0.525
Cardiac output max (l/min)	81	0.56	0.6 (0.2 to 1.1)	0.009**	0.55	0.2 (0.013 to 0.47)	0.039*
Body composition							
Bodyweight (kg)	108	0.97	-0.1 (-0.5 to 0.4)	0.802	0.97	-0.1 (-0.31 to 0.16)	0.370
Fat mass (kg)	111	0.90	-0.03 (-0.5 to 0.4)	0.903	0.91	-0.24 (-0.44 to -0.03)	0.028*
Lean body mass (kg)	110	0.96	0.04 (-0.3 to 0.4)	0.815	0.96	0.1 (-0.03 to 0.3)	0.113
Biomarker							
HbA_1c_ (%)	106	0.51	0.03 (-0.01 to 0.07)	0.128	0.51	0.02 (-0.01 to 0.04)	0.145
Insulin (pmol/l)	106	0.37	-3.1 (-7.8 to 1.6)	0.192	0.44	-4.15 (-6.4 to -2.0)	0.001***
Leptin (ng/ml)	101	0.60	-0.6 (-1.4 to 0.3)	0.193	0.62	-0.56 (-0.97 to -0.15)	0.008**
Quality of Life & Fatigue							
FSS	89	0.53	-0.1 (-0.3 to 0.1)	0.421	0.53	-0.05 (-0.1 to 0.05)	0.306
EORTC	79	0.04	4.2 (0.01 to 8.5)	0.049*	0.002	1 (-1 to 3)	0.324

Abbreviations: MVPA: moderate-to-vigorous intensity physical activity; Beta: unstandardized regression coefficient; VO_2max_: maximum oxygen uptake; HbA1C = glycosylated hemoglobin; EORTC QLQ = European Organization for Research and Treatment of Cancer Quality of Life questionnaire; FSS = Fatigue severity scale; Note: All models (except Body composition Outcomes) were adjusted for age, gender, body mass index and Baseline values. The Body composition Outcomes were adjusted for age, gender, and Baseline values *significant p-value < 0.05; ** highly significant p-value < 0.01; *** very highly significant p-value < 0.001

## Discussion

The main findings from this observational analysis are significant associations between aerobic physical activity (as measured by MVPA or steps), device-based tracking during six months after surgery, and VO_2max_, cardiac output, and metabolic biomarkers (insulin and leptin) in cancer survivors. Our results for MPA (108 min/wk) and VPA (41 min/wk) in combination are within the range of current recommendations for MVPA [5]. Unfortunately, half of the German and U.S. adult population currently does not achieve the physical activity level of 150–300 min/wk MPA [34, 35]. For steps, a large prospective cohort study reported that accruing more steps per day (up to ~ 10000 steps/day) was associated with steady declines in cancer and CVD incidence [36]. The measured physical activity was device-based, but only for one week, and may not represent effective habitual walking behavior. Our findings indicate an average of 8498 per day, which falls within the recommended range of approximately 7.000–10.000 steps per day for cancer patients [37]. It is reasonable to suggest that a practical step threshold for people with chronic disease and lower physical fitness might be below 7.000 steps per day.

Many epidemiological studies have investigated the association between MVPA and systemic health, classifying intensity in METs based on the absolute energy demands of different physical activities [38, 39]. Absolute measures can lead to misclassification of individual exercise intensity, such as moderate or vigorous, because they do not account for factors like body weight and composition, sex, fitness level, or diseases [40]. The amount of self-reported physical activity, which is based on absolute intensity measured in METs, does not consider the individual´s maximum cardiorespiratory fitness or the relative intensity of their exercise. For example, an older person with a low VO_2max_ of approximately 5 METs, working at 4 METs, is exercising at a vigorous intensity (80% of VO_2max_). In contrast, a younger person with a higher VO_2max_ of 15 METs, working at the same absolute intensity of 4 METs, is exercising at a light to moderate intensity (about 33% of their VO_2max_) [41]. For individual exercise prescription, particularly for deconditioned individuals with chronic diseases, a relative measure of intensity — defined as the energy cost of the activity relative to the individual’s maximal capacity — is more suitable [40]. In contrast to other studies involving self-reported questionnaires or device-based recorded physical activity for only 3–7 days, and to the best of our knowledge, this prospective observational cohort analysis is the first to have measured the aerobic physical activity continuous device-based over six months and made a direct association on cardiorespiratory and metabolic systemic outcomes in cancer patients.

At baseline, our participants had a mean VO_2max_ of 27.5 ml/kg/min, which corresponds to a maximum MET value of 7.8. Most wearable devices, like the Garmin vivoactive 4 and 4s, which feature wrist-based heart rate sensors, calculate MVPA by comparing the current heart rate to the average resting heart rate. They utilize the heart rate reserve as the basis for determining relative exercise intensity to estimate MVPA. For moderate (3-5.9 MET) and vigorous (≥ 6 MET), the relative exercise intensity is classified as 40–59% and 60–89% of the heart rate reserve or VO_2_R [40]. Training studies suggest that a minimum effective intensity of 30% VO_2_R is suitable for lower-fit subjects [42].

We found that an additional 100 min of MVPA per week, or taking more than 1,000 steps each day, was associated with a higher VO_2max_ (β = 0.5 or β = 0.27). In a 12-month follow-up of another observational analysis within a randomized controlled trial (RCT), the effect of unsupervised, self-motivated physical activity on VO_2max_ was examined in 357 participants with breast, colon, and prostate cancer after six months [43]. The physical activity measurements were also device-based, but only within a single week. There, a 30-minute increase in MVPA per day was positively associated with higher maximum oxygen uptake of 0.34 ml/kg/min (β = 0.34). However, the test subjects could have artificially adjusted their physical activity within the week. In the CRBP-TS trial, physical activity was tracked for six months, which might have reduced this potential disruptive factor.

Heart failure is a recognized consequence of cancer treatment and can affect QoL and survival [44]. The CRBP-TS study was one of the first trials to measure maximum cardiac output pre- and post-intervention in post-surgery cancer survivors. We observed a positive association between increased physical activity and improved pump function in MVPA and steps. In particular, maximum cardiac output increased by 0.6 l/min for 100 MVPA min more per week.

The sequelae of cancer therapy in cancer survivors on body composition often entail a loss of lean body mass and an increase in fat mass, mainly depending on the type of therapy (chemotherapy, radiotherapy, hormone therapy) [45]. We found a slight inverse association between steps and fat mass, but not for lean body mass. As we did not measure energy or protein intake during this period, it is difficult to interpret these changes in body composition. Obesity has a systemic pro-inflammatory effect and is, therefore, a risk factor for developing cancer [46]. Reducing body fat mass should be a primary goal of cancer prevention and treatment.

Diabetes raises the risk of all-cause mortality among breast, prostate, and colorectal cancer survivors [47]. Higher levels of fasting insulin are known to raise the risk of breast cancer recurrence and more significant overall mortality [48]. Similarly, tumors can use insulin signaling to drive glucose uptake while inducing cell survival and proliferation [49]. Physical activity may well lower breast cancer risk by reducing insulin resistance and other biological mechanisms [50]. Our study findings demonstrate a significant inverse association between physical activity and insulin. With 1,000 more steps per day of physical activity, insulin levels were lower, ranging from − 6.4 to -2.0 pmol/l (95% CI). We also detected a similar inverse association with leptin (-0.97 to -0.15) involving a simultaneous reduction in fat mass in our participants. Increased leptin levels were pronounced in relationship with obesity and are associated with higher cancer risk [51]. Leptin may play a pivotal role in the pathogenesis, development, and metastasis of breast cancer [52]. However, as leptin’s role alone and in its relationship with obesity in cancer development and therapy remains unclear, more research is needed [53].

There is strong evidence that physical activity improves QoL and reduces fatigue in cancer patients and cancer survivors [54, 55]. However, our findings for MVPA or steps could not establish an inverse association with fatigue. Our cancer-related QoL results cannot be evaluated due to the adjusted R-squared value of 0.02.

In this investigation, we relied on the Garmin vívoactive 4 and 4s wearable devices to objectively record physical activity. In general, consumer wearable devices can motivate cancer survivors to be more physically active, and breast cancer survivors found that their wearable devices helped them follow activity guidelines [56]. Most devices, such as the Garmin vívoactive 4 and 4s, estimate MVPA using an algorithm that combines accelerometers and optical heart rate measurements (Photoplethysmography). Although MVPA is probably one of the most commonly used parameters regarding the effects of aerobic physical activity on health, there is a lack of comprehensive meta-analyses on the accuracy, particularly for the sensors used in consumer wearable devices, such as the Garmin vivoactive 4. In addition to the bias inherent in the measurement technology, there is also a lack of validity in the device data generation process. However, most studies define a valid day as a wear-time of at least ten hours within one day and a valid interval as a week with at least three valid days [12]. These recommendations for data generation were exceeded in this study. Although clinicians should be cautious when considering MVPA for clinical and research purposes, despite the different methods employed in studies, there is a consensus that consumer wearable devices are valid for measuring MVPA [20]. In addition to MVPA, steps are often used to evaluate aerobic physical activity. Consumer wearable devices accurately measure steps and heart rates in laboratory-based settings [57]. Steps also demonstrated acceptable accuracy under free-living conditions compared to validated technology (ActiGraph and New-Lifestyles NL-2000i) [58]. Compared to other device-based activity outputs, the Garmin vivoactive 4s also reliably indicates step counts under free-living conditions [19]. The orientation towards the number of steps per day can be a valid and reliable control parameter for consumer wearable devices.

### Strengths and limitations

The present study has three essential strengths. First, the CRBP-TS study employed a prospective design, utilizing high-quality data. Second, we tracked the aerobic physical activity device-based and continuously over six months to establish a valid physical activity behavior. If physical activity data were collected only once at baseline over a one-week period, it might not accurately reflect long-term habitual physical activity behavior. There is limited evidence supporting observation periods for device-based physical activity that last longer than one week. Recommendations suggest that it is crucial to wear activity trackers throughout the study period to ensure that reliable and valid data can be obtained for data analysis [59]. Third, we measured cardiorespiratory and metabolic outcomes at the beginning and end of the observational period to make an association between objective physical activity and systemic health outcomes.

The present study has some limitations. First, wearable devices may be faulty in capturing certain types of physical activity, particularly strength training or swimming. So, the amount of physical activity in our study may have been underestimated. Second, wearable devices do not yet distinguish between continuous and interval exercise involving aerobic activities. The effects on CRF can differ depending on the kind of activity at the same amount of MVPA. Third, wearable device sensors cannot validly capture strength training. Activities such as upper body strength training and cycling, which involve static and intense muscle contractions, can affect blood flow in the forearm as assessed by photoplethysmogram, yielding less accurate results [60]. Since static, intense muscle contractions have a minimal effect on blood flow in the forearm, we anticipate a minor margin of error in our measurements. Future studies on physical activity should record strength training, device-based physical activity via manual entries, and the amount of sedentary time required to acquire a completely objective physical activity profile. Fourth, this study was an observational analysis within an RCT, so we can not make any causal claims. However, this method is not unusual and has been practiced frequently in other studies [61, 62]. Fifth, protein and energy consumption were not quantified, affecting body composition and performance beyond physical activity.

## Conclusion

Higher aerobic physical activity tracked continuously using wearable devices over six months revealed a significant association with various systemic health outcomes in cancer survivors. These outcomes included higher oxygen uptake, cardiac output, while showing lower levels of biomarkers such as insulin and leptin. Our findings offer valuable insights into the aerobic physical activity habits of cancer survivors over a long-term observation period. Using objective and cost-effective measurement methods, such as consumer wearable devices, would help to generate comprehensive activity profiles in future research.

## Data Availability

The datasets generated during the present study can be obtained from the corresponding author on reasonable request.

## Abbreviations

BIA: Bioelectrical impedance analysis
BMI: Body mass index
CI: Confidence interval
CRBP-TS: Colorectal, Breast, and Prostate Cancer - Telemonitoring and Self-management
CRF: Cardiorespiratory fitness
CVD: Cardiovascular disease
DRKS: Deutsches Register Klinischer Studien
ECOG: Eastern Cooperative Oncology Group
EORTC: QlQ-C30 European Organisation for Research and Treatment of Cancer Core Quality of Life Questionnaire
FSS: Fatigue Severity Scale
HbA_1c_: hemoglobin A1c
ICD: International Statistical Classification of Diseases and Related Health Problems
LBM: Lean body mass
MET: Metabolic equivalent of task
MoTrPAC: Molecular Transducers of Physical Activity Consortium
MVPA: Moderate-to-vigorous physical activity
MPA: Moderate physical activity
VPA: Vigorous physical activity
PAOD: Peripheral arterial occlusive disease
QoL: Quality of Life
RPP: Rate pressure product
VO_2max_: maximum oxygen uptake
VO_2_R: oxygen uptake reserve
WHO: World Health Organization

## References

[1] Bray F, Laversanne M, Sung H, Ferlay J, Siegel RL, Soerjomataram I, Jemal A. Global cancer statistics 2022: GLOBOCAN estimates of incidence and mortality worldwide for 36 cancers in 185 countries. CA Cancer J Clin. 2024;74:229–63. 10.3322/caac.21834. 38572751 10.3322/caac.21834

[2] Vainshelboim B, Myers J, Matthews CE. Non-exercise estimated cardiorespiratory fitness and mortality from all-causes, cardiovascular disease, and cancer in the NIH-AARP diet and health study. Eur J Prev Cardiol. 2022;29:599–607. 10.1093/eurjpc/zwaa131. 33624091 10.1093/eurjpc/zwaa131 PMC8489355

[3] Kokkinos P, Faselis C, Samuel IBH, Lavie CJ, Zhang J, Vargas JD, et al. Changes in cardiorespiratory fitness and survival in patients with or without cardiovascular disease. J Am Coll Cardiol. 2023;81:1137–47. 10.1016/j.jacc.2023.01.027. 36948729 10.1016/j.jacc.2023.01.027

[4] Wang Q, Zhou W. Roles and molecular mechanisms of physical exercise in cancer prevention and treatment. J Sport Health Sci. 2021;10:201–10. 10.1016/j.jshs.2020.07.008. 32738520 10.1016/j.jshs.2020.07.008 PMC7987556

[5] Bull FC, Al-Ansari SS, Biddle S, Borodulin K, Buman MP, Cardon G, et al. World health organization 2020 guidelines on physical activity and sedentary behaviour. Br J Sports Med. 2020;54:1451–62. 10.1136/bjsports-2020-102955. 33239350 10.1136/bjsports-2020-102955 PMC7719906

[6] Locasale JW. Diet and exercise in cancer metabolism. Cancer Discov. 2022;12:2249–57. 10.1158/2159-8290.CD-22-0096. 36062923 10.1158/2159-8290.CD-22-0096 PMC9547953

[7] Friedenreich CM, Shaw E, Neilson HK, Brenner DR. Epidemiology and biology of physical activity and cancer recurrence. J Mol Med. 2017;95:1029–41. 10.1007/s00109-017-1558-9. 28620703 10.1007/s00109-017-1558-9 PMC5613065

[8] Groen WG, van Harten WH, Vallance JK. Systematic review and meta-analysis of distance-based physical activity interventions for cancer survivors (2013–2018): we still haven’t found what we’re looking for. Cancer Treat Rev. 2018;69:188–203. 10.1016/j.ctrv.2018.07.012. 30077954 10.1016/j.ctrv.2018.07.012

[9] Garcia DO, Thomson CA. Physical activity and cancer survivorship. Nutr Clin Pract. 2014;29:768–79. 10.1177/0884533614551969. 25335787 10.1177/0884533614551969 PMC4470419

[10] Prince SA, Adamo KB, Hamel ME, Hardt J, Connor Gorber S, Tremblay M. A comparison of direct versus self-report measures for assessing physical activity in adults: a systematic review. Int J Behav Nutr Phys Activity. 2008;5:56. 10.1186/1479-5868-5-56. 10.1186/1479-5868-5-56 PMC2588639 18990237

[11] Lee J. A Meta-analysis of the association between physical activity and breast cancer mortality. Cancer Nurs. 2019;42:271–85. 10.1097/NCC.0000000000000580. 29601358 10.1097/NCC.0000000000000580

[12] Chan A, Chan D, Lee H, Ng CC, Yeo AHL. Reporting adherence, validity and physical activity measures of wearable activity trackers in medical research: A systematic review. Int J Med Inf. 2022;160:104696. 10.1016/j.ijmedinf.2022.104696. 10.1016/j.ijmedinf.2022.104696 35121356

[13] Brickwood K-J, Watson G, O’Brien J, Williams AD. Consumer-Based wearable activity trackers increase physical activity participation: systematic review and Meta-Analysis. JMIR Mhealth Uhealth. 2019;7:e11819. 10.2196/11819. 30977740 10.2196/11819 PMC6484266

[14] Franssen WMA, Franssen, Gregor HLM, Spaas J, Solmi F, Eijnde BO. Can consumer wearable activity tracker-based interventions improve physical activity and cardiometabolic health in patients with chronic diseases? A systematic review and meta-analysis of randomised controlled trials. Int J Behav Nutr Phys Act. 2020;17:57. 10.1186/s12966-020-00955-2. 32393357 10.1186/s12966-020-00955-2 PMC7216601

[15] Nguyen NH, Hadgraft NT, Moore MM, Rosenberg DE, Lynch C, Reeves MM, Lynch BM. A qualitative evaluation of breast cancer survivors’ acceptance of and preferences for consumer wearable technology activity trackers. Support Care Cancer. 2017;25:3375–84. 10.1007/s00520-017-3756-y. 28540402 10.1007/s00520-017-3756-y

[16] Lynch BM, Nguyen NH, Moore MM, Reeves MM, Rosenberg DE, Boyle T, et al. A randomized controlled trial of a wearable technology-based intervention for increasing moderate to vigorous physical activity and reducing sedentary behavior in breast cancer survivors: the ACTIVATE trial. Cancer. 2019;125:2846–55. 10.1002/cncr.32143. 31012970 10.1002/cncr.32143

[17] Alessa HB, Chomistek AK, Hankinson SE, Barnett JB, Rood J, Matthews CE, et al. Objective measures of physical activity and cardiometabolic and endocrine biomarkers. Med Sci Sports Exerc. 2017;49:1817–25. 10.1249/MSS.0000000000001287. 28398945 10.1249/MSS.0000000000001287 PMC5561485

[18] Evenson KR, Spade CL. Review of validity and reliability of Garmin activity trackers. J Meas Phys Behav. 2020;3:170–85. 10.1123/jmpb.2019-0035. 32601613 10.1123/jmpb.2019-0035 PMC7323940

[19] Kastelic K, Dobnik M, Löfler S, Hofer C, Šarabon N, Validity. Reliability and sensitivity to change of three Consumer-Grade activity trackers in controlled and Free-Living conditions among older adults. Sens (Basel). 2021. 10.3390/s21186245. 10.3390/s21186245 PMC8473032 34577457

[20] Gorzelitz J, Farber C, Gangnon R, Cadmus-Bertram L. Accuracy of wearable trackers for measuring Moderate- to Vigorous-Intensity physical activity: A systematic review and Meta-Analysis. J Meas Phys Behav. 2020;3:346–57. 10.1123/jmpb.2019-0072.

[21] Falz R, Thieme R, Tegtbur U, Bischoff C, Leps C, Hillemanns P, et al. CRBP-TS - evaluation of a home-based training and health care program for colorectal, breast, and prostate cancer using telemonitoring and self-management: study protocol for a randomized controlled trial. BMC Sports Sci Med Rehabil. 2021;13:15. 10.1186/s13102-021-00244-w. 33622370 10.1186/s13102-021-00244-w PMC7901214

[22] Falz R, Bischoff C, Thieme R, Tegtbur U, Hillemanns P, Stolzenburg J-U, et al. Effect of home-based online training and activity feedback on oxygen uptake in patients after surgical cancer therapy: a randomized controlled trial. BMC Med. 2023;21:293. 10.1186/s12916-023-03010-6. 37553660 10.1186/s12916-023-03010-6 PMC10408062

[23] Darmochwal S, Bischoff C, Thieme R, Gockel I, Tegtbur U, Hillemanns P, et al. Impact of home-based training and nutritional behavior on body composition and metabolic markers in cancer patients: data from the CRBP-TS study. Front Nutr. 2023;10:1152218. 10.3389/fnut.2023.1152218. 37794972 10.3389/fnut.2023.1152218 PMC10546323

[24] Demark-Wahnefried W, Aziz NM, Rowland JH, Pinto BM. Riding the crest of the teachable moment: promoting long-term health after the diagnosis of cancer. J Clin Oncol. 2005;23:5814–30. 10.1200/JCO.2005.01.230. 16043830 10.1200/JCO.2005.01.230 PMC1550285

[25] Garmin. How Does the Intensity Minutes Feature Work? Garmin Customer Support. 2025. https://support.garmin.com/en-HK/?faq=pNU9nnDzzGAHmEavp9rpY8. Accessed 18 Mar 2025.

[26] Caspersen CJ, Powell KE, Christenson GM. Physical activity, exercise, and physical fitness: definitions and distinctions for health-related research. Public Health Rep. 1985;100:126–31. 3920711 PMC1424733

[27] U.S. Department of Health and human Services. 2008 Physical Activity Guidelines for Americans: Be Active, Healthy, and Happy! 2008.

[28] Morrow JR, Mood DP, Zhu W, Kang M. Measurement and evaluation in human performance. Champaign, IL: Human Kinetics; 2023.

[29] Trappe HJ, Löllgen H. Leitlinien Zur ergometrie. [Guidelines for ergometry. German Society of Cardiology–Heart and Cardiovascular Research]. Z Kardiol. 2000;89:821–31. 10.1007/s003920070190. 11077695 10.1007/s003920070190

[30] Charloux A, Lonsdorfer-Wolf E, Richard R, Lampert E, Oswald-Mammosser M, Mettauer B, et al. A new impedance cardiograph device for the non-invasive evaluation of cardiac output at rest and during exercise: comparison with the direct Fick method. Eur J Appl Physiol. 2000;82:313–20. 10.1007/s004210000226. 10958374 10.1007/s004210000226

[31] Woltjer HH, Bogaard HJ, de Vries PM. The intra- and interobserver variability of impedance cardiography in patients at rest and during exercise. Physiol Meas. 1996;17:171–8. 10.1088/0967-3334/17/3/003. 8870057 10.1088/0967-3334/17/3/003

[32] Branco MG, Mateus C, Capelas ML, Pimenta N, Santos T, Mäkitie A, et al. Bioelectrical impedance analysis (BIA) for the assessment of body composition in oncology: A scoping review. Nutrients. 2023. 10.3390/nu15224792. 38004186 10.3390/nu15224792 PMC10675768

[33] Nolte S, Waldmann A, Liegl G, Petersen MA, Groenvold M, Rose M. Updated EORTC QLQ-C30 general population norm data for Germany. Eur J Cancer. 2020;137:161–70. 10.1016/j.ejca.2020.06.002. 32777715 10.1016/j.ejca.2020.06.002

[34] Richter A, Schienkiewitz A, Starker A, Krug S, Domanska O, Kuhnert R, et al. Health-promoting behaviour among adults in Germany - Results from GEDA 2019/2020-EHIS. J Health Monit. 2021;6:26–44. 10.25646/8553. 35146315 10.25646/8553 PMC8734172

[35] HHS, Physical Activity Guidelines Advisory Committee Scientific Report. 2018: 2018 Physical Activity Guidelines Advisory Committee. Physical Activity Guidelines Advisory Committee Scientific Report. In: Services DoHaH. Washington, D.C.; 2018. 2018.

[36] Del Pozo Cruz B, Ahmadi MN, Lee I-M, Stamatakis E. Prospective associations of daily step counts and intensity with cancer and cardiovascular disease incidence and mortality and All-Cause mortality. JAMA Intern Med. 2022;182:1139–48. 10.1001/jamainternmed.2022.4000. 36094529 10.1001/jamainternmed.2022.4000 PMC9468953

[37] Tudor-Locke C, Craig CL, Aoyagi Y, Bell RC, Croteau KA, de Bourdeaudhuij I, et al. How many steps/day are enough? For older adults and special populations. Int J Behav Nutr Phys Act. 2011;8:80. 10.1186/1479-5868-8-80. 21798044 10.1186/1479-5868-8-80 PMC3169444

[38] Shephard RJ. Absolute versus relative intensity of physical activity in a dose-response context. Med Sci Sports Exerc. 2001;33:419–20. 10.1097/00005768-200106001-00008. S400-18; discussion S. 10.1097/00005768-200106001-00008 11427764

[39] Ainsworth BE, Haskell WL, Herrmann SD, Meckes N, Bassett DR, Tudor-Locke C, et al. 2011 compendium of physical activities: a second update of codes and MET values. Med Sci Sports Exerc. 2011;43:1575–81. 10.1249/MSS.0b013e31821ece12. 21681120 10.1249/MSS.0b013e31821ece12

[40] Garber CE, Blissmer B, Deschenes MR, Franklin BA, Lamonte MJ, Lee I-M, et al. American college of sports medicine position stand. Quantity and quality of exercise for developing and maintaining cardiorespiratory, musculoskeletal, and neuromotor fitness in apparently healthy adults: guidance for prescribing exercise. Med Sci Sports Exerc. 2011;43:1334–59. 10.1249/MSS.0b013e318213fefb. 21694556 10.1249/MSS.0b013e318213fefb

[41] Howley ET. Type of activity: resistance, aerobic and leisure versus occupational physical activity. Med Sci Sports Exerc. 2001;33:S364–9. 10.1097/00005768-200106001-00005. discussion S419. 20. 11427761 10.1097/00005768-200106001-00005

[42] Swain DP, Franklin BA. VO(2) reserve and the minimal intensity for improving cardiorespiratory fitness. Med Sci Sports Exerc. 2002;34:152–7. 10.1097/00005768-200201000-00023. 11782661 10.1097/00005768-200201000-00023

[43] Mazzoni A-S, Helgesen Bjørke AC, Stenling A, Börjeson S, Sjövall K, Berntsen S, et al. The role of Long-Term physical activity in relation to Cancer-Related health outcomes: A 12-Month Follow-up of the Phys-Can RCT. Integr Cancer Ther. 2023;22:15347354231178869. 10.1177/15347354231178869. 37358262 10.1177/15347354231178869 PMC10331773

[44] Armenian SH, Lacchetti C, Barac A, Carver J, Constine LS, Denduluri N, et al. Prevention and monitoring of cardiac dysfunction in survivors of adult cancers: American society of clinical oncology clinical practice guideline. J Clin Oncol. 2017;35:893–911. 10.1200/JCO.2016.70.5400. 27918725 10.1200/JCO.2016.70.5400

[45] Baguley BJ, Dalla Via J, Fraser SF, Daly RM, Kiss N. Effectiveness of combined nutrition and exercise interventions on body weight, lean mass, and fat mass in adults diagnosed with cancer: a systematic review and meta-analysis. Nutr Rev. 2023;81:625–46. 10.1093/nutrit/nuac079. 36206176 10.1093/nutrit/nuac079

[46] Ellulu MS, Patimah I, Khaza’ai H, Rahmat A, Abed Y. Obesity and inflammation: the linking mechanism and the complications. Arch Med Sci. 2017;13:851–63. 10.5114/aoms.2016.58928. 28721154 10.5114/aoms.2016.58928 PMC5507106

[47] Tao H, O’Neil A, Choi Y, Wang W, Wang J, Wang Y, et al. Pre- and Post-diagnosis diabetes as a risk factor for All-Cause and cancer-Specific mortality in breast, prostate, and colorectal cancer survivors: a prospective cohort study. Front Endocrinol (Lausanne). 2020;11:60. 10.3389/fendo.2020.00060. 32132977 10.3389/fendo.2020.00060 PMC7040305

[48] Goodwin PJ, Ennis M, Pritchard KI, Trudeau ME, Koo J, Madarnas Y, et al. Fasting insulin and outcome in early-stage breast cancer: results of a prospective cohort study. J Clin Oncol. 2002;20:42–51. 10.1200/JCO.2002.20.1.42. 11773152 10.1200/JCO.2002.20.1.42

[49] Hopkins BD, Goncalves MD, Cantley LC. Insulin-PI3K signalling: an evolutionarily insulated metabolic driver of cancer. Nat Rev Endocrinol. 2020;16:276–83. 10.1038/s41574-020-0329-9. 32127696 10.1038/s41574-020-0329-9 PMC7286536

[50] Friedenreich CM. Physical activity and breast cancer: review of the epidemiologic evidence and biologic mechanisms. Recent Results Cancer Res. 2011;188:125–39. 10.1007/978-3-642-10858-7_11. 21253795 10.1007/978-3-642-10858-7_11

[51] Wu M-H, Chou Y-C, Chou W-Y, Hsu G-C, Chu C-H, Yu C-P, et al. Circulating levels of leptin, adiposity and breast cancer risk. Br J Cancer. 2009;100:578–82. 10.1038/sj.bjc.6604913. 19223908 10.1038/sj.bjc.6604913 PMC2653731

[52] Gu L, Wang C-D, Cao C, Cai L-R, Li D-H, Zheng Y-Z. Association of serum leptin with breast cancer: A meta-analysis. Med (Baltim). 2019;98:e14094. 10.1097/MD.0000000000014094. 10.1097/MD.0000000000014094 PMC6380739 30702563

[53] Jiménez-Cortegana C, López-Saavedra A, Sánchez-Jiménez F, Pérez-Pérez A, Castiñeiras J, Virizuela-Echaburu JA, et al. Leptin, both bad and good actor in cancer. Biomolecules. 2021. 10.3390/biom11060913. 34202969 10.3390/biom11060913 PMC8235379

[54] Campbell KL, Winters-Stone KM, Wiskemann J, May AM, Schwartz AL, Courneya KS, et al. Exercise guidelines for cancer survivors: consensus statement from international multidisciplinary roundtable. Med Sci Sports Exerc. 2019;51:2375–90. 10.1249/MSS.0000000000002116. 31626055 10.1249/MSS.0000000000002116 PMC8576825

[55] Speed-Andrews AE, Courneya KS. Effects of exercise on quality of life and prognosis in cancer survivors. Curr Sports Med Rep. 2009;8:176–81. 10.1249/JSR.0b013e3181ae98f3. 19584603 10.1249/JSR.0b013e3181ae98f3

[56] Phillips SM, Conroy DE, Keadle SK, Pellegrini CA, Lloyd GR, Penedo FJ, Spring B. Breast cancer survivors’ preferences for technology-supported exercise interventions. Support Care Cancer. 2017;25:3243–52. 10.1007/s00520-017-3735-3. 28470368 10.1007/s00520-017-3735-3 PMC5832636

[57] Fuller D, Colwell E, Low J, Orychock K, Tobin MA, Simango B, et al. Reliability and validity of commercially available wearable devices for measuring steps, energy expenditure, and heart rate: systematic review. JMIR Mhealth Uhealth. 2020;8:e18694. 10.2196/18694. 32897239 10.2196/18694 PMC7509623

[58] Tedesco S, Sica M, Ancillao A, Timmons S, Barton J, O’Flynn B. Validity evaluation of the Fitbit Charge2 and the Garmin vivosmart HR + in Free-Living environments in an older adult cohort. JMIR Mhealth Uhealth. 2019;7:e13084. 10.2196/13084. 31219048 10.2196/13084 PMC6607774

[59] Wood WA, Basch E. From intuition to execution: realizing the potential of wearables in oncology. J Oncol Pract. 2017;13:90–2. 10.1200/JOP.2016.020370. 28972831 10.1200/JOP.2016.020370

[60] Zhang Y, Weaver RG, Armstrong B, Burkart S, Zhang S, Beets MW. Validity of Wrist-Worn photoplethysmography devices to measure heart rate: A systematic review and meta-analysis. J Sports Sci. 2020;38:2021–34. 10.1080/02640414.2020.1767348. 32552580 10.1080/02640414.2020.1767348

[61] Gillis C, Fenton TR, Gramlich L, Sajobi TT, Culos-Reed SN, Bousquet-Dion G, et al. Older frail prehabilitated patients who cannot attain a 400 m 6-min walking distance before colorectal surgery suffer more postoperative complications. Eur J Surg Oncol. 2021;47:874–81. 10.1016/j.ejso.2020.09.041. 33041092 10.1016/j.ejso.2020.09.041

[62] Onerup A, Angenete E, Bock D, Haglind E. Association between self-assessed preoperative level of physical activity and postoperative complications - An observational cohort analysis within a randomized controlled trial (PHYSSURG-C). Eur J Surg Oncol. 2022;48:883–9. 10.1016/j.ejso.2021.10.033. 34742613 10.1016/j.ejso.2021.10.033

